# Src-homology 2 domain-containing tyrosine phosphatase 2 promotes oral cancer invasion and metastasis

**DOI:** 10.1186/1471-2407-14-442

**Published:** 2014-06-16

**Authors:** Hsueh-Chun Wang, Wei-Fan Chiang, Hsin-Hsiu Huang, Ying-Ying Shen, Hung-Che Chiang

**Affiliations:** 1 Department of Medical Research, China Medical University Hospital, 40402 Taichung, Taiwan; 2 China Medical University, 40402 Taichung, Taiwan; 3 Department of Oral & Maxillofacial Surgery, Chi-Mei Medical Center, Liouying, 73657 Tainan, Taiwan; 4 Division of Environmental Health and Occupational Medicine, National Health Research Institutes, No.35, Keyan Road, Zhunan, 35053 Miaoli County, Taiwan; 5 Pathology Core Lab., National Health Research Institutes, 35053 Miaoli, Taiwan; 6 National Environmental Health Research Center, National Health Research Institutes, Miaoli, Taiwan

**Keywords:** Extracellular signal-related kinase, Invasion, Metastasis, Oral cancer, Src-homology 2 domain-containing tyrosine phosphatase 2

## Abstract

**Background:**

Tumor invasion and metastasis represent a major unsolved problem in cancer pathogenesis. Recent studies have indicated the involvement of Src-homology 2 domain-containing tyrosine phosphatase 2 (SHP2) in multiple malignancies; however, the role of SHP2 in oral cancer progression has yet to be elucidated. We propose that SHP2 is involved in the progression of oral cancer toward metastasis.

**Methods:**

SHP2 expression was evaluated in paired oral cancer tissues by using immunohistochemical staining and real-time reverse transcription polymerase chain reaction. Isogenic highly invasive oral cancer cell lines from their respective low invasive parental lines were established using a Boyden chamber assay, and changes in the hallmarks of the epithelial-mesenchymal transition (EMT) were assessed to evaluate SHP2 function. SHP2 activity in oral cancer cells was reduced using si-RNA knockdown or enforced expression of a catalytically deficient mutant to analyze migratory and invasive ability in vitro and metastasis toward the lung in mice in vivo.

**Results:**

We observed the significant upregulation of SHP2 in oral cancer tissues and cell lines. Following SHP2 knockdown, the oral cancer cells markedly attenuated migratory and invasion ability. We observed similar results in phosphatase-dead SHP2 C459S mutant expressing cells. Enhanced invasiveness was associated with significant upregulation of E-cadherin, vimentin, Snail/Twist1, and matrix metalloproteinase-2 in the highly invasive clones. In addition, we determined that SHP2 activity is required for the downregulation of phosphorylated ERK1/2, which modulates the downstream effectors, Snail and Twist1 at a transcript level. In lung tissue sections of mice, we observed that HSC3 tumors with SHP2 deletion exhibited significantly reduced metastatic capacity, compared with tumors administered control si-RNA.

**Conclusions:**

Our data suggest that SHP2 promotes the invasion and metastasis of oral cancer cells. These results provide a rationale for further investigating the effects of small-molecule SHP2 inhibitors on the progression of oral cancer, and indicate a previously unrecognized SHP2-ERK1/2-Snail/Twist1 pathway that is likely to play a crucial role in oral cancer invasion and metastasis.

## Background

Protein tyrosine phosphorylation, under the control of 2 opposing chemical reactions catalyzed by protein tyrosine kinase (PTK) and protein tyrosine phosphatase (PTP), plays a vital role in various cellular functions [1]. Disturbing the balance between PTK and PTP activities leads to aberrant tyrosine phosphorylation, and has been linked to the pathogenesis of many cancers [2]. Therefore, as a key regulator of PTK activity, PTP has been considered a potential drug targets for human cancers. Studies have shown that some PTPs can function as oncogenes, including src-homology 2 domain-containing tyrosine phosphatase 2 (SHP2), which is encoded by tyrosine-protein phosphatase non-receptor type 11 [3-7]. In addition, studies have also identified activate mutants of SHP2 in patients with Noonan syndrome, juvenile myelomonocytic leukemia, acute myelogenous leukemia, and certain types of solid tumor [3,6-8]. SHP2 is a ubiquitously expressed phosphatase that can transduce mitogenic, pro-survival, cell-fate and pro-migratory signals from numerous growth factors, cytokines, and extracellular-matrix receptors [2,9-11].

Most deaths cause by cancer are attributed to metastatic disease. Therefore, the prevention of metastasis has become the focus of clinical attention [12]. In oral cancer, metastasis to cervical lymph nodes or distant organs is the primary prognostic indicator [13-15]. Through the invasion-metastasis cascade, cancer cells can breach to the basement membrane to intravasate and ultimately colonize distant sites, requiring reversible changes in cell-cell and cell-extracellular-matrix (ECM) adherence, destruction of matrix and stromal proteins, and motility [16,17]. Several steps of this process can be executed by cancer cells that activate the epithelial mesenchymal transition (EMT) [18], which is programmed by pleiotropically acting transcriptional factors [19], and predominately controlled by various matrix metalloproteinases (MMPs) [20].

Our understanding of invasion and metastasis remains incomplete; thus, understanding the mechanisms underlying oral cancer invasion and metastasis is crucial for facilitating the development of effective therapeutic strategies against human oral cancer. Although SHP2 represents a promising target in cancer treatment, little is known regarding the role of SHP2 involved in oral cancer development. A recent study suggested that SHP2 influences breast-tumor initiating cells, and enhances breast tumor maintenance and progression [9]. Therefore, we hypothesized that SHP2 is involved in oral cancer invasion and metastasis. We observed that SHP2 promotes the invasion and metastasis in oral cancer, and identified an ERK1/2-Snail/Twist1 pathway mediated by SHP2 that might play a major role in oral cancer invasion and metastasis.

## Methods

### Collection of tissue samples

Twenty-one pairs of primary oral cancer and histologically normal oral mucosa adjacent to the tumors were obtained after surgical resection at Chi-Mei Medical Center, Liouying, Tainan, Taiwan, and stored at -80°C until use. All of the human tissue specimens in this study were processed and used with prior approval from the Chi-Mei Medical Center Institutional Review Board and the National Health Research Institute Institutional Review Board (IRB1000202-R2). Samples containing > 70% tumor cells were selected after microscopic examination of representative tissue sections from each tumor.

### Immunohistochemistry

Immunohistochemistry (IHC) was performed to evaluate SHP2 expression in paraffin-embedded oral squamous cell carcinoma specimens. The slides were stained with a SHP2 antibody (1:200, GeneTex Inc., Irvine, CA, USA) by using an automatic slide stainer BenchMark XT (Ventana Medical Systems), and counterstained with Harris hematoxylin. Two independent pathologies evaluated the slides under a light microscope. Immunoreactivity was classified by estimating the percentage (P) of tumor cells exhibiting characteristic staining (from an undetectable level, 0%, to homogeneous staining, 100%) and by estimating the intensity (I) of staining (1, weak staining; 2, moderate staining; and 3, strong staining). Results were scored by multiplying the percentage of positive cells by the intensity, (i.e. quick score Q = P × I; maximum = 300) [21].

### Real-time reverse transcription-polymerase chain reaction

Real-time reverse transcription-polymerase chain reaction (RT-PCR) analysis of SHP1, SHP2, Snail, Twist1 and GAPDH was conducted using SYBR-Green Master Mix (Roche Applied Science, Basel, Switzerland) according to the manufacturer's instructions. PCR amplifications were performed using an ABI7900 thermal cycler by applying the following amplification conditions: 50°C for 2 min, 95°C for 10 min, for 40 cycles at 95°C for 15 s (denaturation step), and 60°C for 1 min (annealing/extension steps). GAPDH was amplified as an internal control. All of the experiments were performed in duplicate. Relative expression of the target genes (SHP1, SHP2, Snail, and Twist1) to the control gene (GAPDH) was calculated using the ΔC_T_ method: relative expression = 2^-ΔC^_T_, where ΔC_T_ = C_T (Target)_ - C_T (GAPDH)_[22]. The oligonucleotide primers for human SHP1, SHP2, Snail, Twist1, and GAPDH are listed as follows: SHP2, forward: 5’-TCGTATAAATGCTGCTGAAAT-3’, reverse: 5’- TCCTGTTGTTGTAGTGTCT-3’; SHP1, forward: 5’-GCAGTACAAGTTCATCTA-3’, reverse: 5’-CAGGTTCTCATACACATC-3’; Snail, forward: 5’-ACGAGGTGTGACTAACTATG-3’, reverse: 5’-GACAAGTGACAGCCATTAC-3’; Twist1, forward: 5’- TGATAGAAGTCTGAACAGTTGT-3’, reverse: 5’-GCACGACCTCTTGAGAAT-3’; GAPDH, forward:5’-ACACCCACTCCTCCACCTTT-3’, reverse: 5’- AGCCAAATTCGTTGTCATACC-3’.

### Cell culture

HSC3 cells (JCRB, JCRB0623) were provided by Dr. Lu-Hai Wang, Institute of Molecular and Genomic Medicine, National Health Research Institute, Taiwan. The HSC3 cells were cultured in Dulbecco’s modified Eagle’s medium supplemented with 100 μL/mL of fetal bovine serum [23].

### Establishment of highly invasive oral cancer cell lines

The highly invasive HSC3 cell line was established using the Falcon Cell Culture Inserts with a Matrigel coating (BD Biosciences, CA, USA). Briefly, cells (5 × 10^4^) were harvested, re-suspended in a serum-free medium with 0.1-% bovine serum albumin (BSA) (Sigma-Aldrich, Inc., St. Louis, MO, USA), and then plated in a transwell chamber. The chamber was incubated for 18 h with a complete culture medium added to the lower chamber. After 18 h of incubation, cells migrating to the lower surface of the filter were collected [23]. This in vitro selection protocol was used in selecting cells from 4 to 8 cycles to derive the highly invasive sub-lines, HSC3-Inv4 and HSC3-Inv8; in these terms, the number following “Inv” denotes the number of cycles of selection. After invasion selection, the lines were tested for their migratory and invasive ability by performing a Boyden chamber migration/invasion assay [24].

### Cell proliferation assay

Cell viability was measured using the 3-(4, 5-dimethylthiazol-2-yl)-2, 5-diphenyl-2H- tetrazolium bromide (MTT) colorimetric assay. The HSC3 cells were plated at 10^3^ cells/well in a 96-well plate (100 μL/well) and incubated for 24 h. After 24 h, the culture medium was removed, and 200 μL of a fresh medium containing 20 μL of MTT (5 mg/mL; Sigma-Aldrich Japan, Tokyo, Japan) was added to each well. The cells were incubated at 37°C for 4 h. After 4 h, the liquid was discarded and DMSO (200 μL/well) was added, after which the samples were mounted on a micromixer for 15 min to make dissolve the blue granules in the samples thoroughly. The culture plate was then placed on the microplate reader, and optical density (OD) was measured at 570 nm [23].

### SHP2 plasmid construction and transient transfection

Total RNA was isolated from normal human oral keratinocytes (HOK cells) by using the Trizol reagent (Life Technologies, New York, NY, USA). Two microgram aliquots were reverse-transcribed using SuperScript II reverse transcriptase (Life Technologies) and the oligo dT primer according to the manufacturer’s instructions [22]. The human SHP2 coding region (GeneBank: NM_002834) was amplified by performing PCR using the forward primer 5’-GGATCCATGACATCGCGGAGATGGTTT-3’, which introduced a BamHI site, and the reverse primer 5’- GAATTCTTCATCTGAAACTTTTCTGCTG-3’, which introduced an EcoRI site, under the following conditions: denaturing for 30 s at 94°C, annealing for 30 s at 62°C and elongation for 1 min at 72°C for 35 cycles. The full-length of SHP2 was subcloned into the constitutive mammalian expression vector pCMV Tag 2B vector (Stratagene, La Jolla, CA, USA). The SHP2C459S (SHP2C/S) mutant was generated using the QuikChange Lighting Site-Directed Mutagenesis kit (Agilent Technologies, Inc., Wilmington, USA). The HSC3 cells were transfected with the pCMV Tag 2B-SHP2 wild type (WT) or the SHP2C/S mutant and empty vector by using a lipofectamine reagent (Life Technologies), according to the manufacturer’s protocol, and then subjected to invasion, metastasis assays and western blot analysis. The pEGFP-SHP2 WT and C/S mutant were engineered by inserting a coding region into the SalI and BamHI sites of pEGFP vector (Stratagene). The HSC3 cells were transfected with the pEGFP-SHP2 WT or the SHP2 C/S mutant and empty vector, and harvested for use in the immunoprecipitation assay.

### Transfection of cells with siRNA

The HSC3 cells were transfected at 50% confluence with SHP2 siRNA or a scrambled control (Invitrogen Stealth™ RNAi Negative Control LOGC, Life Technologies), Lipofetamine RNAimax (Life Technologies) and Optimen I (Life Technologies) according to the manufacturer's instructions [24]. The RNAi sequences for human SHP2 are listed as follows: SHP2#1, sense: 5’-UAA AUCGGUACUGUGCUUCUGUCUG-3’, antisense: 5’-CAGACAGAAGCACAG ACCGAUUUA-3’; SHP2#2, sense: 5’-AAUAUUUGUAUAUUCGUGCCCUUU C-3’, antisense: 5’- GAA AGG GCACGAAUAUACAAAUAUU-3’. The target sequence for si-RNA is within the SHP2 coding region.

### Assay of SHP2 activity

SHP2 activity was analyzed using a Human Active SHP-2 kit (R&D Systems Inc., Minneapolis, MN, USA). Briefly, cells were lysed in a lysis buffer ([50 mM HEPES, 0.1 mM EGTA, 0.1 mM EDTA, 120 mM NaCl, 0.5-% Nonidet-P40 [NP-40], pH 7.5 supplemented with fresh protease-inhibitor-mixture tablets (Roche Applied Science). The SHP2 proteins were then immunoprecipitated using active SHP2 immunoprecipitation beads (R&D Systems Inc.), and washed 3 times in the lysis buffer and 4 times in a phosphatase assay buffer (10 mM HEPES, 0.1 mM EGTA, 0.1 mM EDTA, 0.5-% BSA, 1 mM dithiothreitol [DTT], pH 7.5). The phosphatase reaction was initiated by incubating the immunocomplexes for 30 min at 37°C in the presence of tyrosine phosphatase substrate I, DADEY (PO3) LIPQQG, according to the manufacturer's instructions. Phosphatase activity was determined using a microplate reader (SpectraMax 190 Absorbance Microplate Reader; Molecular Devices) at 620 nm.

### Western blot analysis

The HSC3 cells were lysed in a RIPA buffer (50 mM Tris–HCl, pH 7.8; 150 mM NaCl; 5 mM EDTA; 5 μL/mL of Triton X-100; 5 μL/mL of NP-40; 1 μL/mL of sodium deoxycholate) and subjected to western blot analysis with the indicated antibodies. The bands were detected and revealed by applying enhanced chemiluminescence (ECL) using ECL western blotting detection reagents and exposed to X-ray film (GE Healthcare, Little Chalfont, Buckinghamshire, UK). Western blot images were captured using an AlphaImager Mini System (Alpha Innotech, Corp., San Leangro, CA, USA) [22]. Detailed antibodies and reagents were described in the Additional file 1.

### Immunoprecipitation

The HSC3 cells were transfected with the pEGFP-SHP2 or the C/S mutant and treated with a lysis buffer (50 mM KP [pH 7.5], 100 mM KCl, 1 mM MgCl_2_, 10-% glycerol, 0.2-% NP-40, 1 mM EGTA, 1 mM NaF, 1 mM sodium pyrophosphate) supplemented with 1 mM DTT, 0.1 mM PMSF, 1 mM sodium orthovanadate and protease inhibitor cocktail tablets (Roche Applied Science). Cell lysates were mixed with an antiserum against Flag, GFP and the immunocomplexes were collected on protein A/G-Sepharose beads (Amersham Pharmacia Biotec) [25]. Western blotting of proteins was performed as described previously.

### Cell migration and invasion assays

The migration and invasion of oral cancer cells were assessed using Falcon Cell Culture Inserts with or without a Matrigel coating (BD Biosciences, CA, USA). Briefly, cells (5 × 10^4^) were harvested, re-suspended in a serum-free medium with 0.1-% BSA (Sigma-Aldrich, Inc., St. Louis, MO, USA), and then plated in a transwell chamber. The chamber was incubated for 18 h with a complete culture medium added to the lower chamber. Cells migrating to the lower chamber were stained with crystal violet. Photomicrographs of 3–5 regions were captured from duplicated chambers and the numbers of cells were counted [26].

### Immunofluorescence staining

The HSC3 cells grown on glass coverslips were fixed with 4-% paraformaldehyde for 10 min, permeabilized with 0.5-% Triton X-100 for 10 min, and blocked with 10-% BSA for 1 h. The cells were then incubated with a rabbit anti-E-cadherin antibody (1:200) for 1 h, before being incubated with FITC-conjugated anti-rabbit immunoglobulin (1:200; Life Technologies) for 30 min. Fluorescence images were captured using a Leica TCS SP5 confocal microscope [27].

### Assay of metastasis

Male CB17/SCID mice (aged 4–6 weeks; 20–25 g) were obtained from BioLASCO Taiwan Co., Ltd and maintained under specific pathogen-free conditions. All experiments were approved by the Animal Care and Use Committee at the National Health Research Institutes, Taiwan (NHRI-IACUC-101117-A). HSC3 cells (1 × 10^5^) were suspended in 100 μM phosphate-buffered saline and injected into the tail vein of mice (4 in each group), before being received control si-RNA (Invitrogen Stealth™ RNAi Negative Control) or SHP2 siRNA (10 μL/g body weight) mixed with the Invivofectamine transfection reagent (Life Technologies) through tail vein injection (100 μL) every 7 d for the next 5 wks. The mice were sacrificed 5 weeks after the injection of HSC3 cells [28-30]. The entire lung was removed, fixed, embedded in paraffin and then sectioned for hematoxylin and eosin (H&E) staining. Tissue images were captured using a Zeiss Mirax Scan 150 microscope (Carl-Zeiss, Oberkochen, Germany). SHP2 siRNA, sense: 5’-UAA AUCGGUACUGUGCUUCUGUCUG-3’, antisense: 5’-CAGACAGAAGCACAG ACCGAUUUA-3’.

### Cellular fractionations

The cytoplasmic and nuclear protein fractions of HSC3 cells were extracted using a NE-PER* Nuclear and Cytoplasmic Extraction Kit (Thermo Fisher Scientific, Yokohama, Japan) according to the manufacturer's instructions [31]. Briefly, cells were harvested in cytosol fractionation buffer supplemented with fresh phosSTOP Phosphatase and Protease Inhibitor Cocktail Tablets (Roche Applied Science) and incubated on ice for 10 min before being centrifuged at 16 000 × *g* for 10 min. The precipitated pellet was solubilized with a nuclear fractionation buffer and then centrifuged at 16000 × g for 10 min.

### MMP-2 secretion assay

A MMP-2 ELISA Kit (EMD Millipore, Inc., Darmstadt, Germany) was used to detect MMP-2 secretion. Briefly, conditioned medium were collected and subjected to an immobilized capture antibody specific for MMP-2. After unbound material was washed away, a synthetic substrate was added to measure absorbance using a spectrophotometric plate reader according to the manufacturer's instructions.

### Statistical analysis

All data were analyzed using the Student’s *t* test and are presented as the mean ± SD. Difference were considered to be statistically significant at *P *< 0.05*.

## Results

### Upregulation of SHP2 expression correlates with the migratory and invasive ability of oral cancer cells

**Figure 1:**
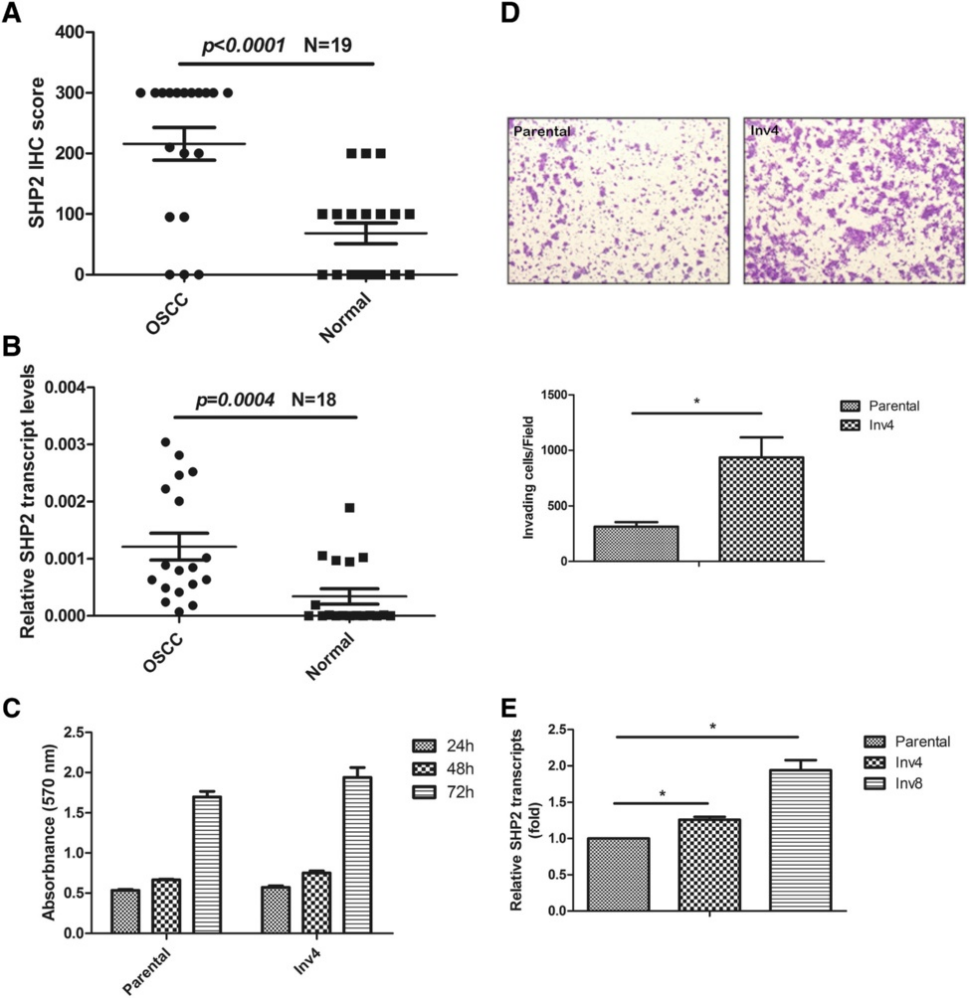
**Upregulation of SHP2 expression correlates with the migratory and invasive ability of oral cancer cells. (A)** Oral tumors and histologically normal oral mucosa adjacent to the tumors were stained with anti-SHP2 antibody. The IHC semi-quantitative score was derived by two independent pathologies, multiplying the staining intensity by the percent of tumor cells stained. IHC scores for each core of a specimen were averaged (n = 19) and statistically analyzed. **(B)** cDNA from paired oral tumor samples were subjected to RT-PCR (n = 18). Relative expression of SHP2 transcript to internal control gene, GAPDH was calculated as described in Materials and Methods. **(C)** Cell proliferation was performed by MTT assay. Cells were counted at 570 nm wavelength and the relative absorbance was represented as mean ± SD from at least four independent experiments. **(D)** Cells were seeded onto the transwell chamber coated with matrigel as described in Methods. Images are representative of cells adhering to the lower chamber after the invasive process. Cells were stained with crystal violet solution, and images were taken by photography (Upper panel). Invading cells per file on the lower chamber were counted. The data are expressed as mean ± SD from three independent experiments; P *< 0.05*. (Lower panel) **(E)** An increased SHP2 transcript level was associated with higher invasive ability of HSC3 cells. The expression of SHP2 for HSC3-Inv4 and HSC3-Inv8 was normalized to HSC3 parental cells.

To assess the potential role of SHP2 in oral tumorigenesis, we evaluated SHP2 expression in human oral tumors, and paired and histologically normal oral mucosa adjacent to the tumors. We subjected 2 type tissue samples to IHC staining for SHP2 and observed a significantly higher SHP2 in tumor cells than in histologically normal oral mucosa adjacent to the tumors (Figure 1A). Real-time quantitative RT-PCR analysis supported these results and indicated significantly higher levels of the SHP2 transcript in tumor tissue than in histologically normal oral mucosa adjacent to the tumors (Figure 1B).

To investigate the biological functions of SHP2 in oral tumorigenesis, we isolated highly invasive clones from oral cancer cells by using an in vitro invasion assay. We used 4–8 cycles of HSC3 cells, which have modest migratory and invasive ability among oral cancer cell lines (data not shown), to derive the highly invasive clones, HSC3-Inv4 and HSC3-Inv8. The growth of these clones was the same as that of the parental cells (Figure 1C), but the number of HSC3-Inv4 cells that migrated through the filter was significantly higher than the number of parental cells that migrated through the filter (Figure 1D). We observed significantly upregulated SHP2 expressions in the HSC3-Inv4 and HSC3-Inv8 clones in comparison with the parental cells (Figure 1E). We observed no significant difference in the levels of the SHP1 transcript in the clones and parental cells (Additional file 2: Figure S1). SHP1 is a high homolog of SHP2. Therefore, these results suggested that SHP2 may exclusively be responsible for the migration and invasion of oral cancer cells.

### SHP2 activity is required for the migration and invasion of oral cancer cells

**Figure 2:**
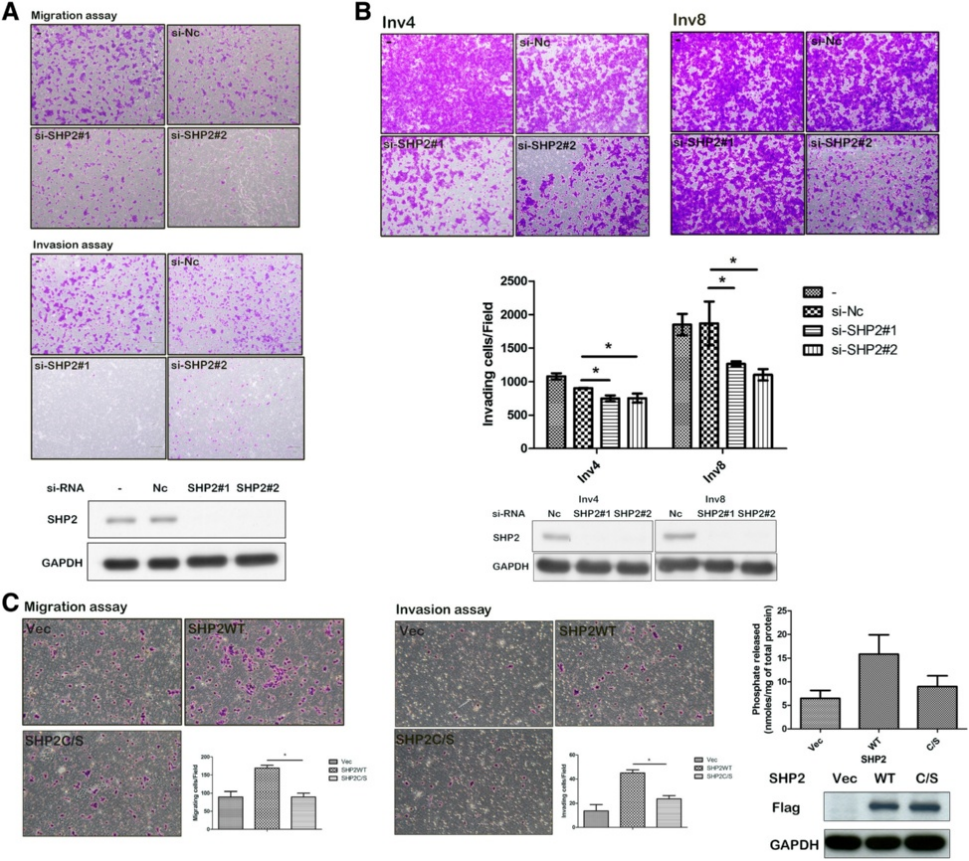
**SHP2 depletion or catalytic deficiency mutant inhibits migration and invasion of oral cancer cells. (A)** Cells transfected with SHP2 si-RNA (si-SHP2#1 or si-SHP2#2) or Negative control (si-NC) were seeded onto the transwell chamber coated with or without matrigel as described in Materials and Methods. Cells adhering to the lower chamber after the migration or invasive process were stained with crystal violet solution, and images were taken under bright-field microscopy at 40×. An obvious decrease in migration (Upper panel) and invasion (middle panel) ability was noted in HSC3 cells transfected with SHP2 si-RNA (si-SHP2#1 or si-SHP2#2) compared to Negative control (si-NC). Western blot shows the expression of SHP2 in HSC3 cells transfected with SHP2 si-RNA or Negative control (Lower panel). **(B)** Effect of SHP2 knockdown on invasion of HSC3-Inv4 and HSC3-Inv8 cells (Upper panel, left and right, respevtively). The quantitative data are expressed as mean ± SD from three independent experiments; *, P < 0.05 (Middle panel). Western blot shows the expression level of SHP2 in HSC3-Inv4 and HSC3-Inv8 cells transfected with SHP2 si-RNA or Negative control (Lower panel, left and right, respectively). **(C)** A dramatic decrease in migration (Left panel) and invasion ability (Middle panel) was observed in HSC3 cells transfected with SHP2 C459S mutant (SHP2C/S) compared to the SHP2 wild type (SHP2WT). Evaluation on SHP2 activity of the cells transfected with indicated constructs. Experiments were done in triplicate at least, and values are indicated as mean ± SD. *, P < 0.05 (Right upper panel). Western blot shows the expression level of transfected flag-SHP2 proteins (Right lower panel).

To determine whether SHP2 is involved in regulating oral cancer migration and invasion, we knocked down SHP2 by using specific si-RNA. As expected, when we downregulated SHP2 expression, the oral cancer cells exhibited markedly reduced migratory and invasive ability (Figure 2A). We observed similar effects on the invasive ability of the HSC3-Inv4 and HSC3-Inv8 cells (Figure 2B). Collectively, our results indicated that SHP2 plays a crucial role in migration and invasion in oral cancer cells.Considering the crucial role of SHP2 activity in various cellular functions, we then investigated whether SHP2 activity is required for migration and invasion of oral cancer cells. We generated a flag-tagged SHP2 WT or phosphatase-dead SHP2 C459S mutant in HSC3 cells. When we analyzed the cell migration or invasion, we observed that the SHP2 mutant abrogated cell migration and invasion elicited by the SHP2 WT (Figure 2C). Overall, these data indicated that the catalytic activity of SHP2 is required for the migration and invasion of oral cancer cells.

### Critical events associated with enhanced invasiveness in oral cancer cells

**Figure 3:**
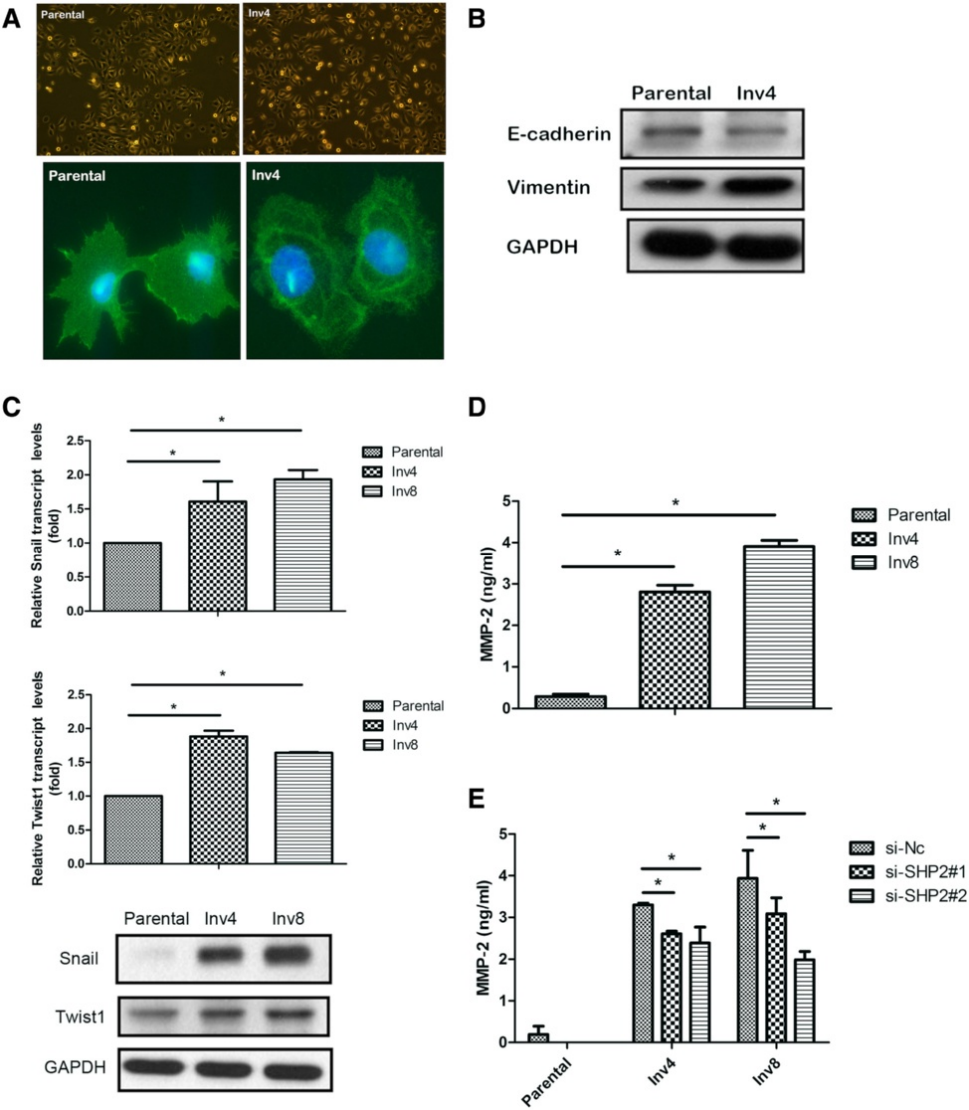
**Characteristics of highly invasive clone, HSC3-Inv4 derived from parental HSC3 cells. (A)** Bright file microscopy images of HSC3 parental and HSC3 Inv 4 (20×, Upper panels). Cells were stained with E-cadherin and images were taken under fluorescence at 60× (Lower panels). **(B)** Expressions of E-cadherin and vimentin were analyzed by Western blot with indicated antibodies; GAPDH as a loading control. **(C)** Increased Snail (Upper panel) and Twist1 (Middle panel) transcript levels were observed in HSC3-Inv4 and HSC3-Inv8 compared to HSC3 parental cells. Experiments were done at least in triplicate and values indicated as mean ± SD. *, P *< 0.05* compared with the adjacent normal in each case. Western blot shows the expression level of Snail and Twist1 in HSC3-parental, HSC3-Inv4 and HSC3-Inv8 cells (Lower panel). **(D)** Status of MMP-2 secretion on highly invasive clones. Medium collected from HSC3 parental, HSC-Inv4 and HSC3-Inv8 cells were subjected to MMP-2 secretion analysis. Significantly increased amounts of MMP-2 were seen in selected sub-cell lines compared to parental cells. **(E)** SHP2 depletion resulted in decreased MMP-2 secretion in HSC3 parental, HSC3-Inv4 and HSC3-Inv8 cells.

As shown in Figure 3A, we evaluated the changes in EMT-associated E-cadherin and vimentin in highly invasive oral cancer cells. Our results indicated that the majority of the parental HSC3 cells were polygonal in shape (Figure 3A, left upper panel); whereas, the HSC3-Inv4 cells were rather spindle shaped (Figure 3A, right upper panel), with downregulated of E-cadherin protein and upregulated of vimentin protein (Figure 3B). When we evaluated the levels of the transcripts of EMT regulators Snail/Twist1, we observed significant upregulation of Snail/Twist1 mRNA expression levels in the highly invasive clones generated from the HSC3 cells (Figure 3C).We then tested the medium from the highly invasive clones to evaluate the secretion of MMP-2. As shown in Figure 3D, increased MMP-2 secretion from oral cancer cells significantly correlated with increased cell invasion. While we analyzed the medium from SHP2-depleted cells, we observed significantly reduced MMP-2 (Figure 3E). Collectively, these results suggested that SHP2 exerts its function in several critical stages that contribute to the acquirement of invasiveness during oral cancer metastasis.

### SHP2 regulates Snail/Twist1 expression through ERK1/2 signaling

**Figure 4:**
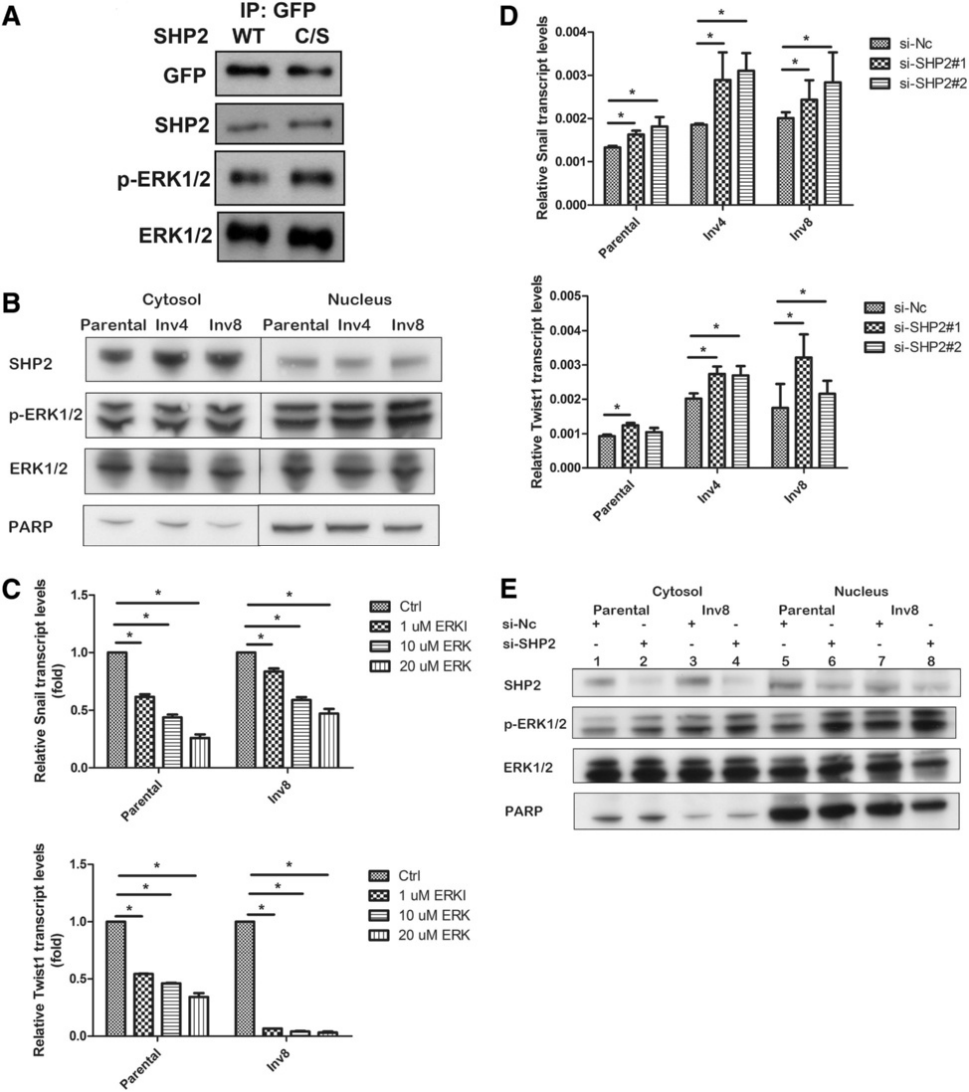
**SHP2 acts on Snail/Twist1 through negatively regulating ERK1/2 activity. (A)** SHP2 forms a complex with ERK1/2. Total cell lysates were prepared, and SHP2 was immunoprecipitated from HSC3 cells expressing EGFP-tagged SHP2 wild type or catalytic-defective SHP2 (SHP2C/S). SHP2 in association with active ERK1/2 in these cells was detected by SDS-PAGE and immunoblotting with anti-phospho-ERK1/2, ERK1/2, SHP2 and GFP. **(B)** Nuclear localization of phospho-ERK1/2 is enriched in HSC3-Inv4 and HSC3-Inv 8 compared to HSC3 parental cells. **(C)** Treatment of ERK inhibitor with indicated concentration for 6 hours significantly reduced Snail or Twist1 mRNA expression in HSC3 parental and HSC3-Inv8 cells. **(D)** SHP2 depletion significantly increased Snail orTwist1 mRNA expression in HSC3 parental and HSC3-Inv8 cells (Upper panel and lower panel, respectively.). Experiments were done in triplicate and values indicated as mean ± SD. *, P *< 0.05* compared with adjacent normal in each case. **(E)** Knockdown of SHP2 increases both cytosol and nuclear localization of phospho-ERK1/2 in oral cancer cells. Poly ADP-ribose polymerase (PARP) was used as a nuclear marker.

To identify the potential biochemical pathways that rely on SHP2 activity, we analyzed total tyrosine phosphorylation in SHP2 WT- and C459S mutant-expressing cells. As shown in Additional file 3: Figure S2, we observed increased protein phosphorylation in mutant-expressing cells, particularly those migrating around 40–50 kD on the gel, compared with SHP2 WT-expressing cells. We thus hypothesized that p44/42 (ERK1/2) signaling might trigger nuclear events because the phosphorylation of ERK1/2 leads to its translocation to the nucleus, which is required for the induction of several cellular responses. By immunoprecipitating exogenously expressed EGFP-tagged SHP2 and immunoblotting using anti-ERK1/2 as a probe, we identified an association between ERK1/2 and SHP2 in cells expressing SHP2 WT and mutant (Figure 4A). We observed markedly increased ERK1/2 phosphorylation in phosphatase-dead cells (Figure 4A), indicating that SHP2 catalytic activity plays a major role in the regulation of ERK1/2 activity, but is not required for the assembly of the ERK1/2/SHP2 complex.Considering the hypothesis that increased ERK1/2 phosphorylation leads to its accumulation in the nucleus (Figure 4B), we then investigated whether Snail and Twist1 are possible downstream effectors of ERK1/2 signaling. In the presence of a selective ERK1/2 inhibitor, FR180204, we observed a dose-dependent reduction at the transcript levels of Snail/Twist1 in oral cancer cells (Figure 4C). However, in the absence of SHP2 expression, we observed increased transcript levels of Snail/Twist1 (Figure 4D), as well as increased ERK1/2 phosphorylation (Figure 4E). These results supported that SHP2 modulates Snail/Twist1 at a transcript level by negatively regulating ERK1/2 activity.

### SHP2-depleted oral cancer cells exhibit reduced ability for lung metastasis

**Figure 5:**
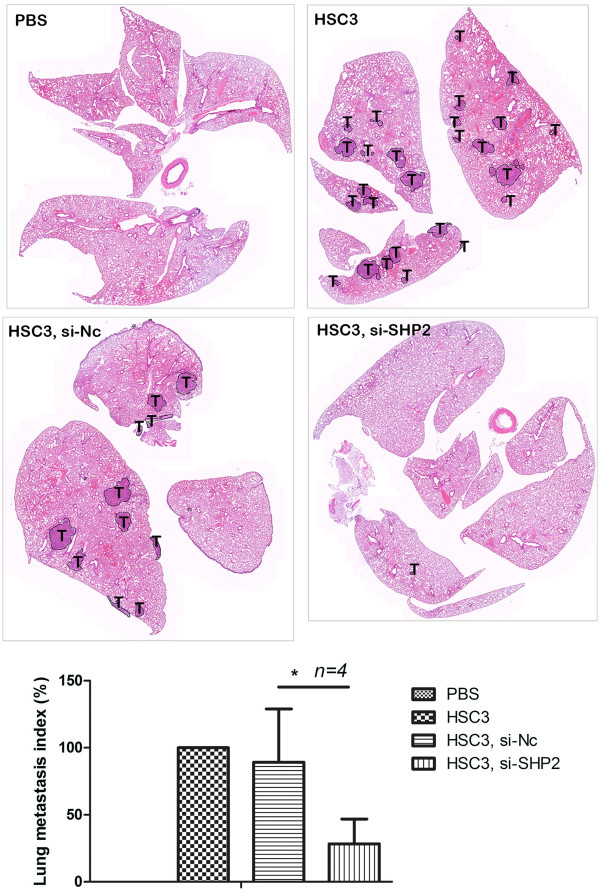
**SHP2 promotes lung metastasis.** SHP2 si-RNA delivered via tail vein injection dramatically reduced the metastatic capacity of HSC3 cells. Representative images showing H&E staining of lung tissues were taken under bright-field at 200× using a scanning microscope (Upper panel). Black lines delineate tumor tissue (T). Quantitative metastasis index was indicated as mean ± SD. *, P *< 0.05* compared with the control group, HSC3 cells (Lower panel).

We evaluated the effects of SHP2 attention on the metastasis of oral cancer cells toward the lung to establish the potential for developing SHP2 as a target for human oral cancer treatment. As shown in Figure 5, we analyzed the lungs of mice with HSC3 xenografts and SHP2 si-RNA administered through tail vein injection by using H&E staining. Analysis of lung tissue sections indicated that HSC3 tumors with SHP2 knockdown exhibited an approximate 70% reduction in metastatic capacity, compared with those with control si-RNA (Figure 5, lower panel). Overall, the result supported that SHP2 inhibits the migration, invasion, and metastasis of oral cancer cells, and indicated that SHP2 is a potential target for oral cancer treatment.

## Discussion

Studies have reported that SHP2 is overexpressed and/or hyperactive in multiple malignancies [3,4,6,7,24,32]; however, the role of SHP2 in oral cancer has yet to be elucidated fully. Our results indicated that the levels of SHP2 transcript (Figure 1A) and SHP2 protein (Figure 1B) were significantly upregulated in tissue samples obtained from patients with oral cancer, and that SHP2 is required for the in vitro invasion of oral cancer cells to Matrigel (Figure 2A and B) and in vivo metastasis of oral cancer cells toward the lung in mice (Figure 5). Considering the requirement of SHP2 activity for the migration and invasion of oral cancer cells (Figure 2C), and the significant upregulation of SHP2 activity in oral cancer cells (Additional file 4: Figure S3), we investigated whether SHP2 mutations cause the observed increase in SHP2 activity in oral cancer cells. We did not identify any SHP2 mutations in oral cancer cell lines and tissue samples (data not shown), supporting the findings of previous studies that SHP2 mutations rarely occur in solid tumors [3,9,32]. Therefore, SHP2 hyperactivity in oral cancer cells might result from the inappropriate expression of SHP2 binding protein, which causes the aberrant activation of SHP2 [33,34]. However, additional studies are required to confirm this hypothesis.

In the study, we isolated highly invasive oral cancer cell clones to establish useful method for investigating the mechanisms underlying the invasion and metastasis of oral cancer cells. We evaluated critical stages in invasion-metastasis cascade, including EMT and MMPs (Figure 3). Previous studies have reported reduced E-cadherin expression in oral cancer cells with highly invasive ability, and we observed similar results in this study. The methylation of E-cadherin might cause the downregulation of E-cadherin expression, which plays a major role in invasion and metastasis in oral cancer. Recent studies have also shown that Snail-dependent EMT in oral cancer cells occurs as a result of the downregulation of E-cadherin [35], and that Twist1, another important transcriptional factor involved in the EMT, was upregulated in cells isolated from patients with metastatic oral squamous cell carcinoma [36]. The highly invasive clones also exhibited changes in the hallmarks of the EMT and transcriptional factors responsible for the EMT, providing a suitable cell model for the analysis of the detailed mechanisms involved in oral cancer metastasis. Our results indicated that SHP2 increases MMP-2 secretion in oral cancer cells (Figure 3E). Previous studies have suggested that the ERK1/2 pathway increases the invasion of several cancers by increasing MMP-2/9 expression and activity [37-40]. However, treatment of the oral cancer cells with ERK inhibitor resulted in no significant changes in MMP-2 secretion (data not shown), indicating that signaling pathways other than ERK1/2 might be involved in SHP2-mediated MMP-2 secretion.

Our results suggest a mechanism which SHP2 downregulates ERK1/2 activity and, thus, regulates Snail/Twist1 expression (Figure 4). The downregulation of epidermal growth factor receptor activity by SHP2 might downregulate ERK1/2 signaling (Additional file 5: Figure S4). However, the interaction between SHP2 and ERK1/2 in oral cancer cells suggests that the effects of SHP2 on ERK1/2 activity occur through direct or indirect interaction between the enzymes (Figure 4A). Therefore, the interaction partners of SHP2 in oral cancer cells must be investigated to elucidate the detailed mechanisms underlying the effects of SHP2 on ERK1/2 regulation. The functional consequences of SHP2-ERK1/2-Snail/Twist1 signaling have yet to be established. SHP2-mediated Snail/Twist1 regulation through ERK1/2 may not be critical to the EMT. Alternatively, Snail/Twist1 may be involved in steps other than the EMT during oral cancer progress. Additional studies are required to evaluate these hypotheses.

Because no selective SHP2 inhibitor was available, we used a specific SHP2 si-RNA to evaluate the role of SHP2 in the metastasis of oral cancer cells toward the lung in mice (Figure 5). PTPs have increasingly attracted attention as targets for novel cancer therapies. Our in vivo si-RNA knockdown data indicated that SHP2 siRNA can be applied in patients with oral cancer. Studies have indicated that SHP2 is responsible for the basal suppression of pSTAT1 and subsequent antigen processing machinery component-mediated immune escape in head and neck cancer cells [24], suggesting that SHP2 can be targeted to enhance T-cell-based cancer immunotherapy. Overall, these findings emphasize the potential use of SHP2 as a treatment target for oral cancer.

## Conclusions

In this study, we report that SHP2 is a potential target for oral cancer treatment. We overexpressed SHP2 in oral cancer cells, and attenuated SHP2 to observe reduced invasion and metastasis. Our result indicated that the downregulatory effects of SHP2 on ERK1/2 might regulate Snail/Twist1 mRNA expression and play a crucial role in oral cancer invasion and metastasis. These findings provide a rationale for future investigation into the effects of small-molecule SHP2 inhibitors on oral cancer progression, and can facilitate the development of novel treatments for human oral cancer.

## Abbreviations

ERK: extracellular signal-related kinase; PARP: Poly ADP-ribose polymerase; SHP2: Src-homology 2 domain-containing tyrosine phosphatase 2.

## Competing interests

No potential conflicts of interest were disclosed.

## Authors’ contributions

HCW designed the study, conducted experiments, analyzed and interpreted data and wrote the manuscript. WFC ensured protocol integrity and collected data. HHH conducted experiments and collected data. YYS analyzed and interpreted data. HCC reviewed the manuscript. All authors read and approved the final manuscript.

## Pre-publication history

The pre-publication history for this paper can be accessed here:

http://www.biomedcentral.com/1471-2407/14/442/prepub

## Supplementary Material

Additional file 1 Suplemetary materials and Methods. Click here for file

Additional file 2: Figure S1 SHP1 transcriptional level is not associated with highly invasive ability in oral cancer cells. No significant difference in SHP1 transcript was observed between parent and highly invasive clones derived from HSC3 cells. The expression of SHP1 for HSC3-Inv4 and HSC3-Inv8 was normalized to HSC3 parental cells. Data are representative of three independent experiments. Click here for file

Additional file 3: Figure S2 SHP2 catalytic-defective expressing cells showed enhanced tyrosine phosphorylation of protein. The cells expressing SHP2 wild type or C/S mutant were lysed, and subjected to immunoblotting with anti-phospho-tyrosine. Data are representative of three independent experiments. Click here for file

Additional file 4: Figure S3 Profile of SHP2 activity in oral cancer cell lines (OC3, OECM1, HSC3, and SCC4). Experiments were done in triplicate at least, and values are indicated as mean ± SD. HOK, normal cells. Click here for file

Additional file 5: Figure S4 SHP2 negatively regulates EGFR activity in oral cancer cells. Total cell lysates were prepared, and SHP2 was immunoprecipitated from HSC3 cells expressing EGFP-tagged SHP2 wild type or catalytic-defective SHP2 (SHP2C/S). SHP2 in association with active EGFR in these cells was detected by SDS-PAGE and immunoblotting with anti-phospho-EGFR, EGFR, and SHP2. GAPDH as loading control. Data are representative of three independent experiments. Click here for file
