# Supplementary material for: Src-homology 2 domain-containing tyrosine phosphatase 2 promotes oral cancer invasion and metastasis
Source: BMC Cancer. 2014 Jun 16;14:442. doi: 10.1186/1471-2407-14-442 (PMC4067087; doi:10.1186/1471-2407-14-442)
Supplement: Additional file 1 — Suplemetary materials and Methods. [file 1471-2407-14-442-S1.docx]

**Supplementary Materials and Methods**

**Cell Culture**

Human normal human oral keratinocytes, HOK, and OC3, OECM1 and SCC4 were cultured as previously described [[1](#_ENREF_1)].

**Antibodies and reagents**

Anti-flag antibody was purchased from Sigma-Aldrich, Inc. Anti-GAPDH antibody was purchased from Santa Cruz Biotechnology, Inc. (Santa Cruz, CA, USA). Anti-E-cadherin, anti-phospho-EGFR, anti-EGFR, anti-phospho-ERK1/2, anti-ERK1/2, anti-SHP2, anti-Snail and anti-Vimentin antibodies were purchased from Cell Signaling Technology, Inc. (Boston, MA, USA). Anti-phosphotyrosine (4G10) and anti-PARP antibodies were purchased from Merck Millipore. Anti-GFP and anti-Twist1 antibodies were purchased from GeneTex Inc. (Irvine, CA, USA). ERK inhibitor II, FR180204 was purchased from EMD Millipore.

**References**

1. Wang HC, Chiang WF, Huang HH, Huang SK, Chiang HC: Promoter hypermethylation of the gene encoding heat shock protein B1 in oral squamous carcinoma cells. Oral surgery, oral medicine, oral pathology and oral radiology 2013, 115(3):376-384.
