# Supplementary figures and images for: Src-homology 2 domain-containing tyrosine phosphatase 2 promotes oral cancer invasion and metastasis
Source: BMC Cancer. 2014 Jun 16;14:442. doi: 10.1186/1471-2407-14-442 (PMC4067087; doi:10.1186/1471-2407-14-442)

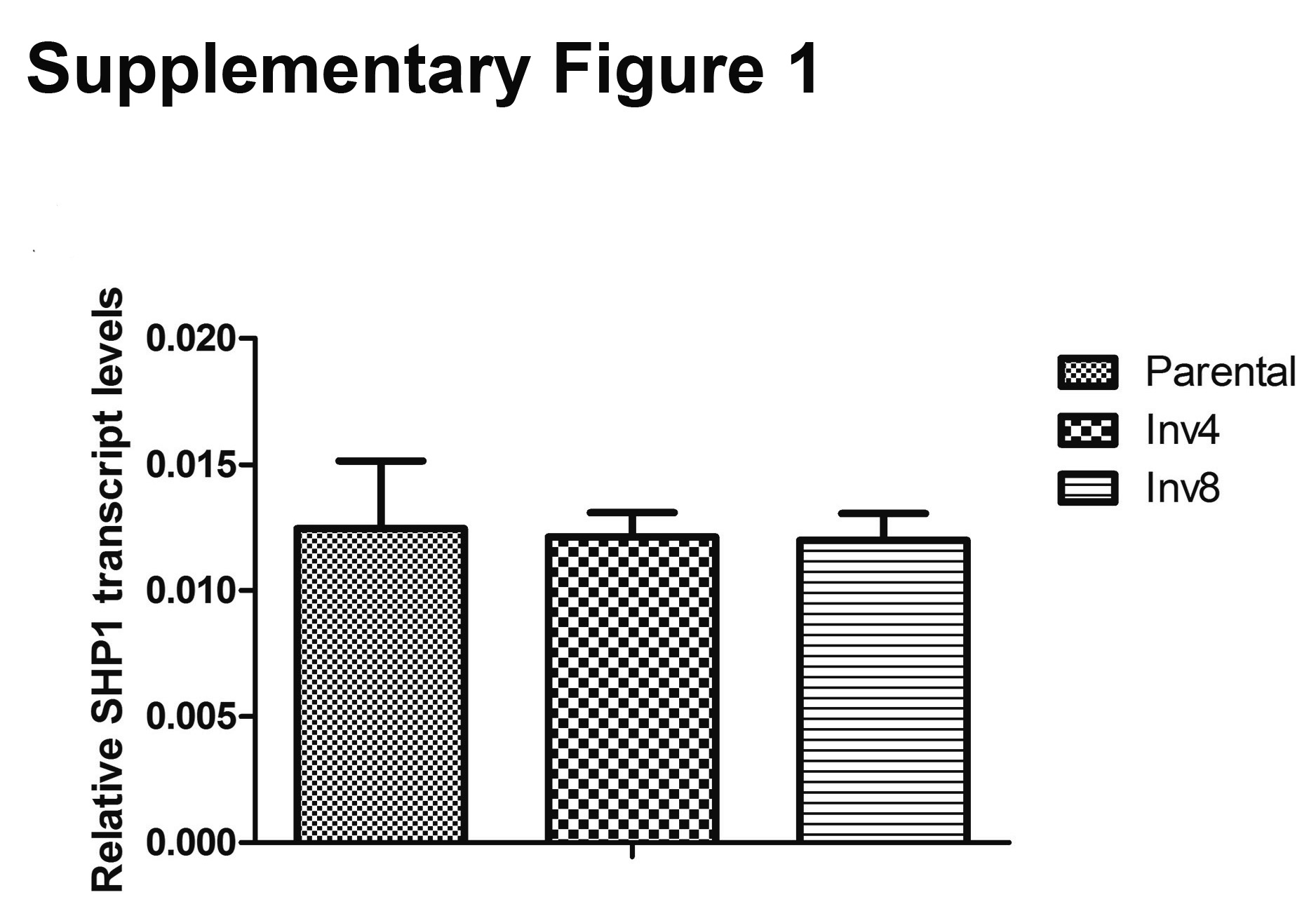

Supplement: Additional file 2: Figure S1 — SHP1 transcriptional level is not associated with highly invasive ability in oral cancer cells. No significant difference in SHP1 transcript was observed between parent and highly invasive clones derived from HSC3 cells. The expression of SHP1 for HSC3-Inv4 and HSC3-Inv8 was normalized to HSC3 parental cells. Data are representative of three independent experiments. [file 1471-2407-14-442-S2.jpeg]

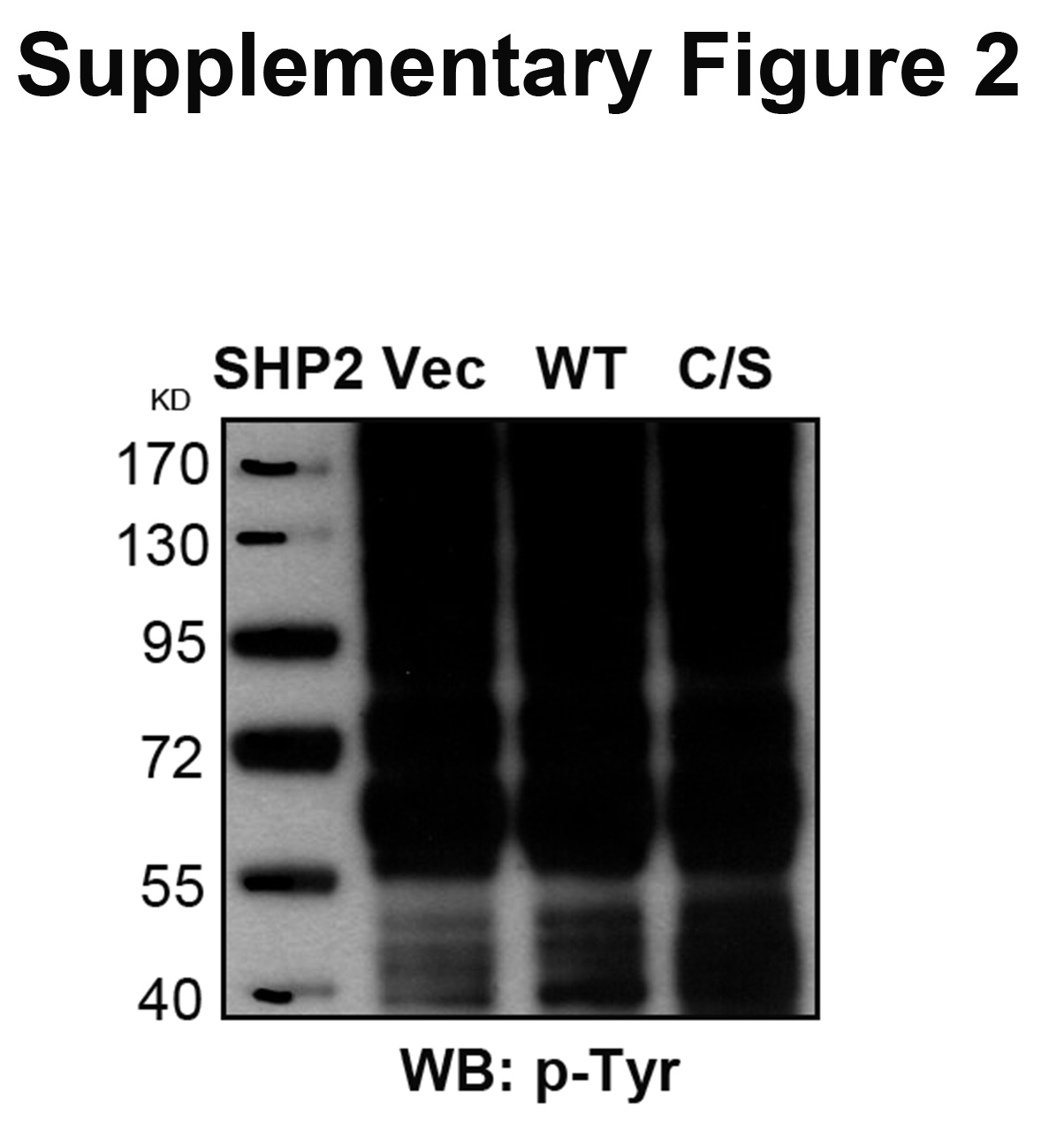

Supplement: Additional file 3: Figure S2 — SHP2 catalytic-defective expressing cells showed enhanced tyrosine phosphorylation of protein. The cells expressing SHP2 wild type or C/S mutant were lysed, and subjected to immunoblotting with anti-phospho-tyrosine. Data are representative of three independent experiments. [file 1471-2407-14-442-S3.jpeg]

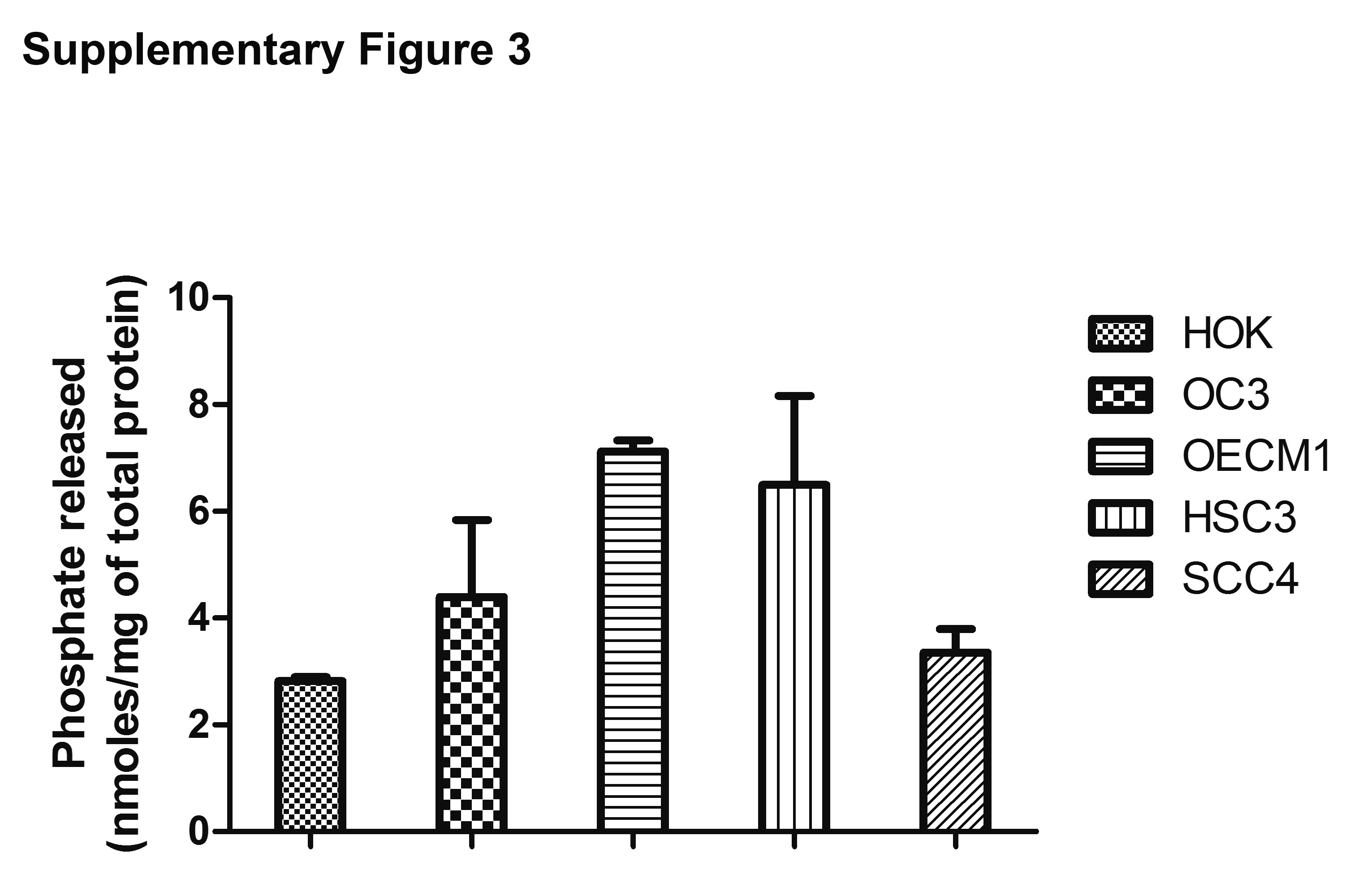

Supplement: Additional file 4: Figure S3 — Profile of SHP2 activity in oral cancer cell lines (OC3, OECM1, HSC3, and SCC4). Experiments were done in triplicate at least, and values are indicated as mean ± SD. HOK, normal cells. [file 1471-2407-14-442-S4.jpeg]

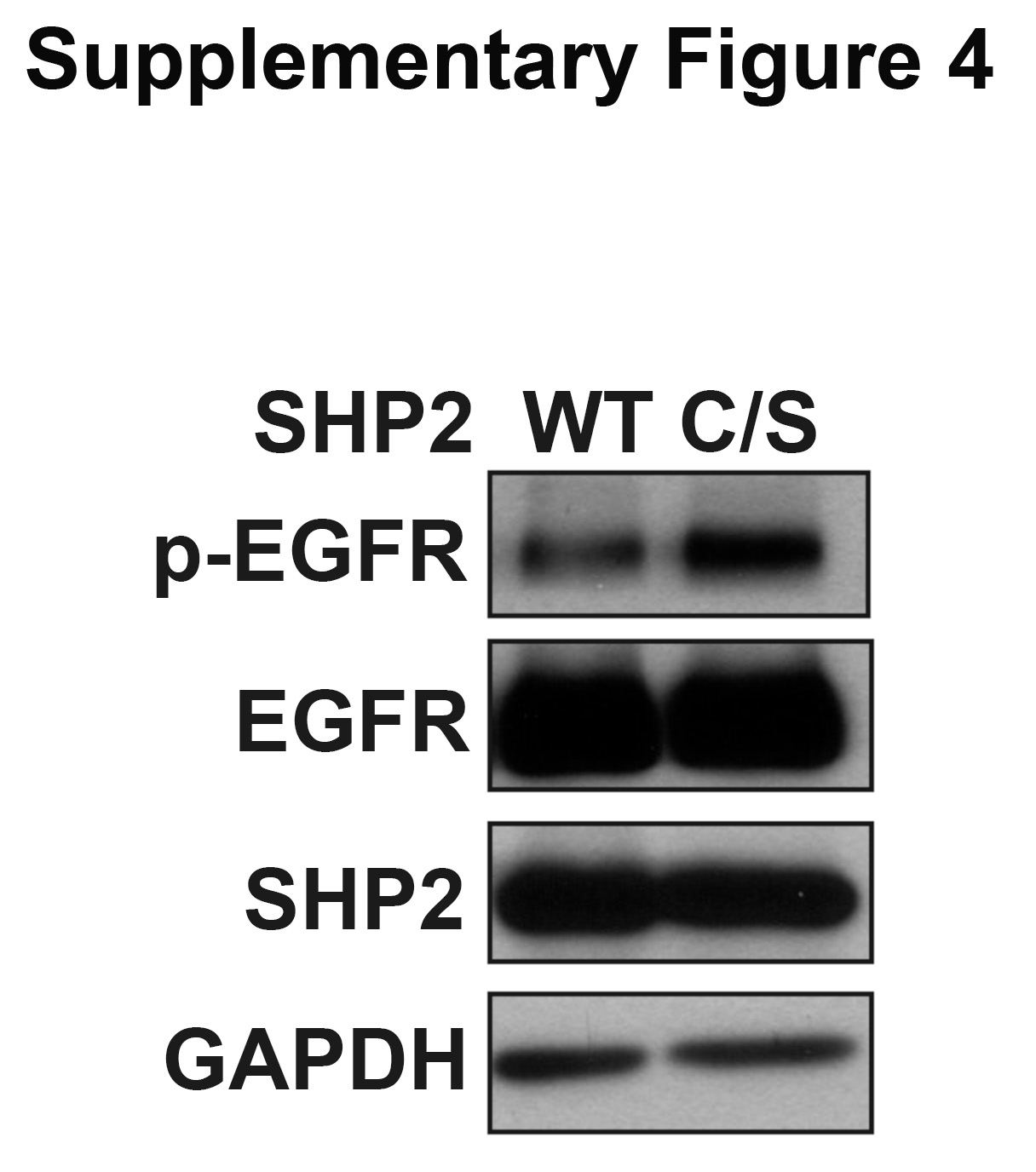

Supplement: Additional file 5: Figure S4 — SHP2 negatively regulates EGFR activity in oral cancer cells. Total cell lysates were prepared, and SHP2 was immunoprecipitated from HSC3 cells expressing EGFP-tagged SHP2 wild type or catalytic-defective SHP2 (SHP2C/S). SHP2 in association with active EGFR in these cells was detected by SDS-PAGE and immunoblotting with anti-phospho-EGFR, EGFR, and SHP2. GAPDH as loading control. Data are representative of three independent experiments. [file 1471-2407-14-442-S5.jpeg]
